# Research on the Role of Marriage Status Among Women Underwent Breast Reconstruction Following Mastectomy: A Competing Risk Analysis Model Based on the SEER Database, 1998–2015

**DOI:** 10.3389/fsurg.2021.803223

**Published:** 2022-01-21

**Authors:** Siyuan Zhang, Zejian Yang, Pei Qiu, Juan Li, Can Zhou

**Affiliations:** ^1^Department of Breast Surgery, First Affiliated Hospital, Xi'an Jiaotong University, Xi'an, China; ^2^School of Medicine, Xi'an Jiaotong University, Xi'an, China; ^3^Department of Translational Medicine Center, First Affiliated Hospital of Xi'an Jiaotong University, Xi'an, China

**Keywords:** marital status, breast reconstruction, competing risk model analysis, SEER, breast cancer

## Abstract

**Background:**

Marital status is an important foundation of social public relations in modern society, but little is known about the role of marriage status among women who underwent breast reconstruction following mastectomy. This research mainly aimed to investigate the prognostic value of marital status in breast cancer women who underwent breast reconstruction.

**Methods:**

The demographic and clinical data of patients were obtained from the Surveillance, Epidemiology, and End Results (SEER) Program database. The eligible population was assessed on overall survival (OS), breast cancer-specific survival (BCSS), and breast cancer-specific death (BCSD) through propensity score matching (PSM) method, multivariate Cox proportional hazards model analysis, competing risk model analysis, multivariate competing risk regression model analysis, and subgroup analysis.

**Results:**

Of the 54,683 women included in the current study, a total of 38,110 participants were married patients (married group), and 16,573 participants were unmarried patients (unmarried group). Patients in the married group tended to have better OS (hazard ratio [HR] = 1.397, 95% CI: 1.319–1.479, *p* < 0.001), BCSS (HR = 1.332, 95% CI: 1.244–1.426, *p* < 0.001), cumulative BCSD incidence (Gray's test, *p* < 0.001), and other causes-specific death (OCSD) incidence (Gray's test, *p* < 0.001) than those in the unmarried group. In subgroup analysis, subjects with HR+/HER2– subtype breast cancer in the married group showed improved OS (1.589, 95% CI: 1.363–1.854, *p* < 0.001) and BCSS (HR = 1.512, 95% CI: 1.255–1.82, *p* < 0.001) than those in the unmarried group.

**Conclusions:**

Our study demonstrated that the inexistence of marriage was associated with poorer OS and BCSS, especially for HR+/HER2– breast cancer women who underwent breast reconstruction.

## Introduction

Since Halsted's radical mastectomy was firstly described in 1882, the awareness and innovations in the reconstructive technique of breast reconstruction have evolved slowly, due to the improving understanding of the biology of breast cancer, paradigmatic evolution in oncologic (1). In addition, both immediate and delayed breast reconstruction have experienced a gradual rise over the past few decades. The popularity of this approach is illustrated by an upsurge in the proportion of breast reconstruction from 8% in 1995 to about 41% in 2013, of which the majority largely consists of prosthetic reconstructions (2, 3). Breast reconstruction means the maintenance of life quality through restoring form, self-perception, and psychosocial and aesthetic benefits, without affecting the long-term prognosis or detection of locoregional recurrence of cancer through restoring the mound of the breast (4). For these reasons, it would be essential to investigate and identify the factors predicting the long-term tumor survival and prognosis of breast reconstruction.

Many studies focused on the impact of clinicopathological features, such as molecular phenotype and Tumor-Node-Metastasis (TNM) stage, and socioeconomic factors, such as quality of social support and marital status, on the survival, development, and prognosis of patients with breast reconstruction (5–8). Among them, marital status has been attached great importance to the important social and psychological factors affecting the long-term prognosis of breast cancer patients (9, 10). However, few randomized trials or well-performed studies have been conducted to study the efficacy of marital status in patients with breast reconstruction. The precise clinical value of marital status for patients with breast reconstruction is still unknown. For these reasons, more studies are urgently needed to confirm the real-world effect of marital status in patients with breast reconstruction.

To further investigate and explore the efficacy of marital status for patients with breast reconstruction, we followed a large cohort of breast cancer women with breast reconstruction based on the population-based database Surveillance, Epidemiology, and End Results (SEER) cancer registry program (11). Statistical methods, such as the Kaplan-Meier method, Cox proportional hazards model, and competing risk regression, were performed to further evaluate the efficiency of marital status on the long-term survival through comparing married patients with unmarried cases who underwent reconstructed breast. This study is expected to provide guidance on the overall survival (OS) for clinicians and breast cancer patients with breast reconstruction through important variables affecting prognosis and facilitating the decision-making of follow-up treatment.

## Materials and Methods

### Patients

**Figure 1:**
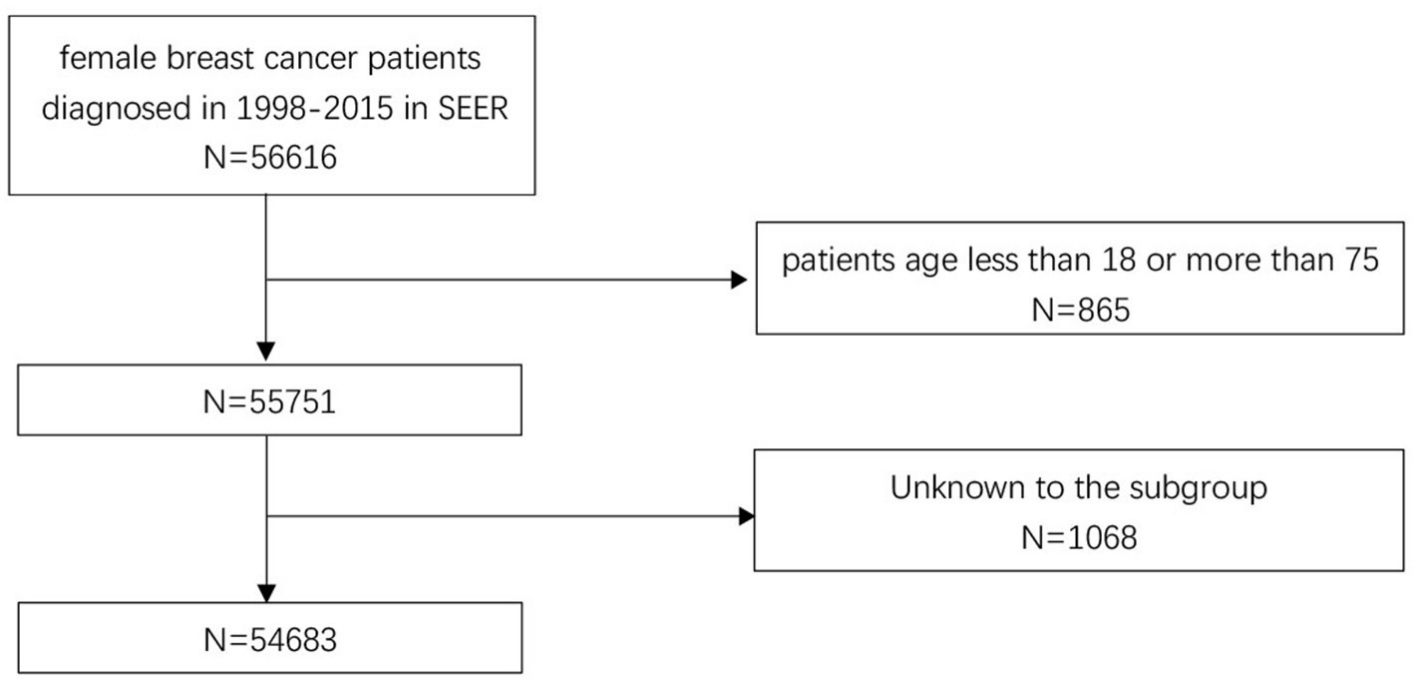
Eligibility, inclusion, and exclusion criteria of the study population.

The data of participants included in this study are obtained from The SEER Program. The SEER program maintained by the National Cancer Institute, is an authoritative source of information on cancer incidence and survival in the United States and collects detailed information on demographics and clinical characteristics from population-based cancer registries, encompassing ~34.6% of the US population (12). In the current study, female patients diagnosed in 1998–2015 with non-metastatic breast carcinoma were first included. Then a series of screening criteria for the patients initially included were performed, the details are shown as a flow diagram in Figure 1. Totally, 54,683 patients were included in this study (ICD-O-3:8470/3, 8480/3, 8481/3,8500/3, 8501/3, 8502/3, 8503/3, 8504/4, 8507/3, 8510/3, 8513/3, 8514/3, 8520/3, 8521/3, 8522/3, 8523/3, 8524/3, 8530/3, 8540/3, 8541/3, 8543/3, 8550/3, 8560/3, 8562/3, 8570/3, 8571/3, 8572/3, 8573/3, 8574/3, and 8575/3). To evaluate the effect of marital status on prognosis, the study cohort was classified into two groups by the existence of marriage: married group and unmarried group. Divorced, widowed, and separated status in marriage were considered as unmarried. “No radiation and/or cancer-directed surgery” was seemed as no radiotherapy. “No/Unknown” chemotherapy recodes was seemed as no chemotherapy.

Demographic characteristics, such as age at diagnosis, race, and marital status, were included. Age at diagnosis was divided into 18–50 years and 51–75 years subgroups. The race included the White race, the Black race, and other race subgroups. The clinical-pathological data included laterality of tumors, histological grade, T stage, N stage, TNM stage, estrogen receptor (ER), progesterone receptor (PR), human epidermal growth factor 2 (HER-2) status. The treatment factors included surgical methods, reconstruction methods, and radiotherapy and chemotherapy status.

### Statistical Analysis

To eliminate the influence of different characteristics between participants in married and unmarried groups, the propensity score matching (PSM) method was utilized to match one unmarried patient with one married patient using the following characteristics: age at diagnosis, race, laterality of tumors, histological grade, T stage, N stage, TNM stage, ER, PR, and HER-2 status, surgical methods, reconstruction methods, and radiotherapy and chemotherapy status. The PSM method was performed by IBM SPSS 25.0 software.

Pearson's χ^2^ test was adopted to compare the demographic characteristics, clinical-pathological data, and treatment factors between the married and unmarried groups. Kaplan-Meier method was performed to generate the survival curve, and the differences between married and unmarried groups were analyzed by log-rank test. The Pearson's χ^2^ test and Kaplan-Meier method were performed by GraphPad Prism 7. The multivariate Cox proportional hazards model analyses were performed to assess the effect of covariates on OS, and hazard ratios (HRs), their corresponding 95% CI. The covariates, such as demographic characteristics, clinical-pathological parameters, and treatment data, were included. The Cox proportional hazards model was conducted with SPSS version 25.0, and the related forest plots were created with the survminer package in R software (version 4.0.2).

Then, to further determine the influence of marital status in different molecular subtypes, the population enrolled was classified into four subgroups (HR+/HER2 +, HR+/HER2–, HR–/HER2–, and HR–/HER2 +) on the basis of molecular typing. The differences between patients in the married and unmarried groups were analyzed by the Kaplan-Meier method and the HRs were reported.

We classified the cause of death as breast cancer-specific death (BCSD) and other causes-specific death (OCSD). The covariates included age at diagnosis, race, marital status, side, grade, tumor size, ER status, PR status, contralateral prophylactic mastectomy (CPM) or not, prophylactic mastectomy or not, mastectomy, reconstruction methods, and radiotherapy and chemotherapy status. The Fine and Gray competing risk model was used to mitigate the estimation bias by classifying death causes into two subgroups. The cumulative incidence function (CIF) and Gray's test were performed to identify and assess the differences of statistical probability due to any competing risk events. The statistical analyses were carried out in the R software (version 4.0.2) using the R package cmprsk (13). A two-sided *p* < 0.05 was considered as statistically significant.

## Results

### Demographics and Clinical Characteristics of Patients

The baseline clinical characteristics of the included patients are shown in Table 1. Of the 54,683 women included in the current study, a total of 38,110 participants were married patients (married group), and 16,573 participants were unmarried patients (unmarried group). Among these women, 27,404 (50.11%) of those were aged 51 or older, 45,751 (83.67%) of those were White race, and 27,404 (50.11%) of those were diagnosed with left breast cancer. In total, 24,381 (44.59%) patients were moderately differentiated (grade II), 44,031 (80.52%) patients were ER positive (ER +), and 38,626 (70.64%) patients were PR positive (PR +). A total of 33,565 (61.28%) participants received a simple mastectomy, 23,562 (43.09%) cases received CPM, 22,193 (40.58%) cases received autologous tissue reconstruction, 11,694 (21.39%) cases received radiotherapy, and 30,949 (56.60%) cases received chemotherapy. By comparing patients in the married and unmarried groups, significant differences (*p* < 0.05) were found in age at diagnosis, race, grade, T stage, N stage, TNM stage, ER status, PR status, molecular subtypes, prophylactic mastectomy, and chemotherapy treatment subgroups.

**Table 1:** Characteristics of married and unmarried patients.

**Characteristics**	**Before PSM**							**After PSM**						
	**Total patients** ***n*** **=** **54,683 (%)**		**Patients married** ***n*** **=** **38,110 (%)**		**patients unmarried** ***n*** **=** **16,573 (%)**		***P* value**	**Total patients** ***n*** **=** **22,838 (%)**		**Patients married** ***n*** **=** **11,419 (%)**		**Patients unmarried** ***n*** **=** **11,419 (%)**		***P* value**
Age at diagnosis							<0.0001							1.00
18–50	27,279	0.4989	19,594	0.5141	7,685	0.4637		10,352	0.4533	5,176	0.4533	5,176	0.4533	
51–75	27,404	0.5011	18,516	0.4859	8,888	0.5363		12,486	0.5467	6,243	0.5467	6,243	0.5467	
Race							<0.0001							1.00
White	45,751	0.8367	32,841	0.8617	12,910	0.7790		20,538	0.8993	10,269	0.8993	10,269	0.8993	
Black	4,954	0.0906	2,303	0.0604	2,651	0.1600		1,396	0.0611	698	0.0611	698	0.0611	
Others	3,978	0.0727	2,966	0.0778	1,012	0.0611		904	0.0396	452	0.0396	452	0.0396	
Side							0.2448							1.00
Left	27,404	0.5011	19,036	0.4995	8,368	0.5049		11,562	0.5063	5,781	0.5063	5,781	0.5063	
Right	27,279	0.4989	19,074	0.5005	8,205	0.4951		11,276	0.4937	5,638	0.4937	5,638	0.4937	
Grade							<0.0001							1.00
I	9,985	0.1826	7,023	0.1843	2,962	0.1787		4,378	0.1917	2,189	0.1917	2,189	0.1917	
II	24,381	0.4459	17,155	0.4501	7,226	0.4360		10,714	0.4691	5,357	0.4691	5,357	0.4691	
III + IV	20,317	0.3715	13,932	0.3656	6,385	0.3853		7,746	0.3392	3,873	0.3392	3,873	0.3392	
T stage							<0.0001							1.00
T0 + T1	31,236	0.5712	22,219	0.5830	9,017	0.5441		13,936	0.6102	6,968	0.6102	6,968	0.6102	
T2	18,221	0.3332	12,402	0.3254	5,819	0.3511		7,550	0.3306	3,775	0.3306	3,775	0.3306	
T3 + T4	5,226	0.0956	3,489	0.0916	1,737	0.1048		1,352	0.0592	676	0.0592	676	0.0592	
N stage							0.0007							1.00
N0	33,579	0.6141	23,526	0.6173	10,053	0.6066		15,602	0.6832	7,801	0.6832	7,801	0.6832	
N1	15,228	0.2785	10,613	0.2785	4,615	0.2785		5,450	0.2386	2,725	0.2386	2,725	0.2386	
N2	5,876	0.1075	3,971	0.1042	1,905	0.1149		1,786	0.0782	893	0.0782	893	0.0782	
TNM stage							<0.0001							1.00
0 + 1	23,745	0.4342	16,899	0.4434	6,846	0.4131		11,452	0.5014	5,726	0.5014	5,726	0.5014	
2	22,751	0.4161	15,706	0.4121	7,045	0.4251		9,034	0.3956	4,517	0.3956	4,517	0.3956	
3	8,187	0.1497	5,505	0.1445	2,682	0.1618		2,352	0.1030	1,176	0.1030	1,176	0.1030	
ER							0.0042							1.00
Positive	44,031	0.8052	30,809	0.8084	13,222	0.7978		19,042	0.8338	9,521	0.8338	9,521	0.8338	
Negative	10,652	0.1948	7,301	0.1916	3,351	0.2022		3,796	0.1662	1,898	0.1662	1,898	0.1662	
PR							0.0009							
Positive	38,626	0.7064	27,082	0.7106	11,544	0.6966		17,360	0.7601	8,680	0.7601	8,680	0.7601	1.00
Negative	16,057	0.2936	11,028	0.2894	5,029	0.3034		5,476	0.2398	2,739	0.2399	2,737	0.2397	
HER-2							0.283							1.00
Positive	5,356	0.1768	3,724	0.1784	1,632	0.1733		1,488	0.1186	744	0.1186	744	0.1186	
Negative	24,939	0.8232	17,152	0.8216	7,787	0.8267		11,056	0.8814	5,528	0.8814	5,528	0.8814	
Subtype							0.0014							1.00
HR+/HER2–	21,608	0.7133	14,955	0.7164	6,653	0.7063		9,712	0.7742	4,856	0.7742	4,856	0.7742	
HR+/HER2 +	3,784	0.1249	2,626	0.1258	1,158	0.1229		1,096	0.0874	548	0.0874	548	0.0874	
HR–/HER2 +	1,572	0.0519	1,098	0.0526	474	0.0503		392	0.0313	196	0.0313	196	0.0313	
HR–/HER2-	3,331	0.1100	2,197	0.1052	1,134	0.1204		1,344	0.1071	672	0.1071	672	0.1071	
Mastectomy							0.0837							1.00
Simple mastectomy	33,565	0.6138	23,483	0.6162	10,082	0.6083		14,864	0.6508	7,432	0.6508	7,432	0.6508	
Radical mastectomy	21,118	0.3862	14,627	0.3838	6,491	0.3917		7,974	0.3492	3,987	0.3492	3,987	0.3492	
Prophylactic mastectomy							<0.0001							1.00
No	31,121	0.5691	21,291	0.5587	9,830	0.5931		13,414	0.5874	6,707	0.5874	6,707	0.5874	
Yes	23,562	0.4309	16,819	0.4413	6,743	0.4069		9,424	0.4126	4,712	0.4126	4,712	0.4126	
Reconstruction method							0.8587							1.00
Tissue	22,193	0.4058	15,496	0.4066	6,697	0.4041		9,226	0.4040	4,613	0.4040	4,613	0.4040	
Implant	24,402	0.4462	16,984	0.4457	7,418	0.4476		10,776	0.4718	5,388	0.4718	5,388	0.4718	
Combined	8,088	0.1479	5,630	0.1477	2,458	0.1483		2,836	0.1242	1,418	0.1242	1,418	0.1242	
Radiation							0.9007							1.00
No	42,989	0.7861	29,966	0.7863	13,023	0.7858		19,324	0.8461	9,662	0.8461	9,662	0.8461	
Yes	11,694	0.2139	8,144	0.2137	3,550	0.2142		3,514	0.1539	1,757	0.1539	1,757	0.1539	
Chemotherapy							0.0331							1.00
No	23,734	0.4340	16,427	0.4310	7,307	0.4409		11,324	0.4958	5,662	0.4958	5,662	0.4958	
Yes	30,949	0.5660	21,683	0.5690	9,266	0.5591		11,514	0.5042	5,757	0.5042	5,757	0.5042	
Dead							<0.0001							<0.0001
Alive	49,037	0.8968	34,528	0.9060	14,509	0.8755		20,579	0.9011	10,390	0.9099	10,189	0.8923	
BCSD	4,003	0.0732	2,590	0.0680	1,413	0.0853		1,485	0.0650	693	0.0607	792	0.0694	
Other reason	1,643	0.0300	992	0.0260	651	0.0393		774	0.0339	336	0.0294	438	0.0384	

After the PSM, a total of 22,838 subjects were included, of which 11,419 were married and 11,419 were not, and no differences were noted in terms of the covariates abovementioned. Key methodological characteristics before and after PSM are shown in Table 1.

### Marital Status and Survival Analysis

**Figure 2:**
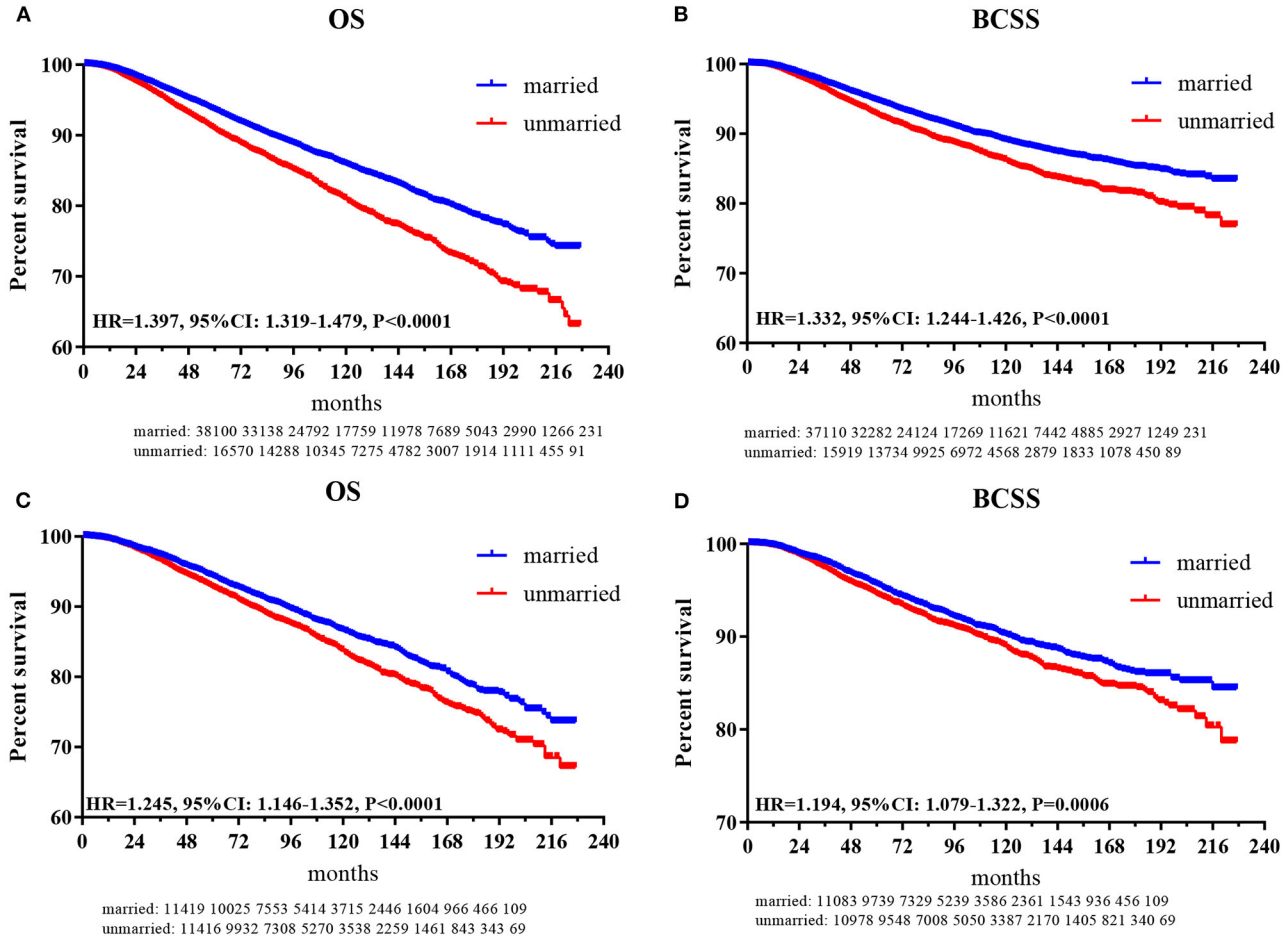
Kaplan-Meier plot to compare married and unmarried patients. **(A)** Overall survival for patients before PSM; **(B)** breast cancer-specific survival for patients before PSM; **(C)** overall survival for patients after PSM; **(D)** breast cancer-specific survival for patients after PSM.

In total, 5,646 (10.32%) subjects died in this study with a median follow-up of 72 months (range, zero to 227 months). The cumulative OS rates at 3-, 5-, 8-, and 10 years for patients in the married group were 96.7, 93.5, 88.7, and 85.8%, respectively, while 95.4, 91.8, 85, and 80.9% for patients in the unmarried group. The cumulative breast cancer-specific survival (BCSS) rates at 3-, 5-, 8-, and 10 years for patients in the married group were 97.3, 94.7, 91.0, and 89.0%, respectively, while 96.4, 92.7, 88.6, and 86.1% for patients in the unmarried group. As shown in Figures 2A,B, the HRs of 1.397 (95% CI: 1.319–1.479, *p* < 0.001) and 1.332 (95% CI: 1.244–1.426, *p* < 0.001) demonstrated that marriage could confer OS and BCSS advantage for female patients with breast reconstruction.

After PSM, as shown in Figures 2C,D, the HRs of 1.245 (95% CI: 1.146–1.352, *p* < 0.001) and 1.194 (95% CI: 1.079–1.322, *p* < 0.001) indicated that marriage could still confer OS and BCSS benefit for female patients with breast reconstruction.

### Multivariate Cox Regression Model Analysis

**Figure 3:**
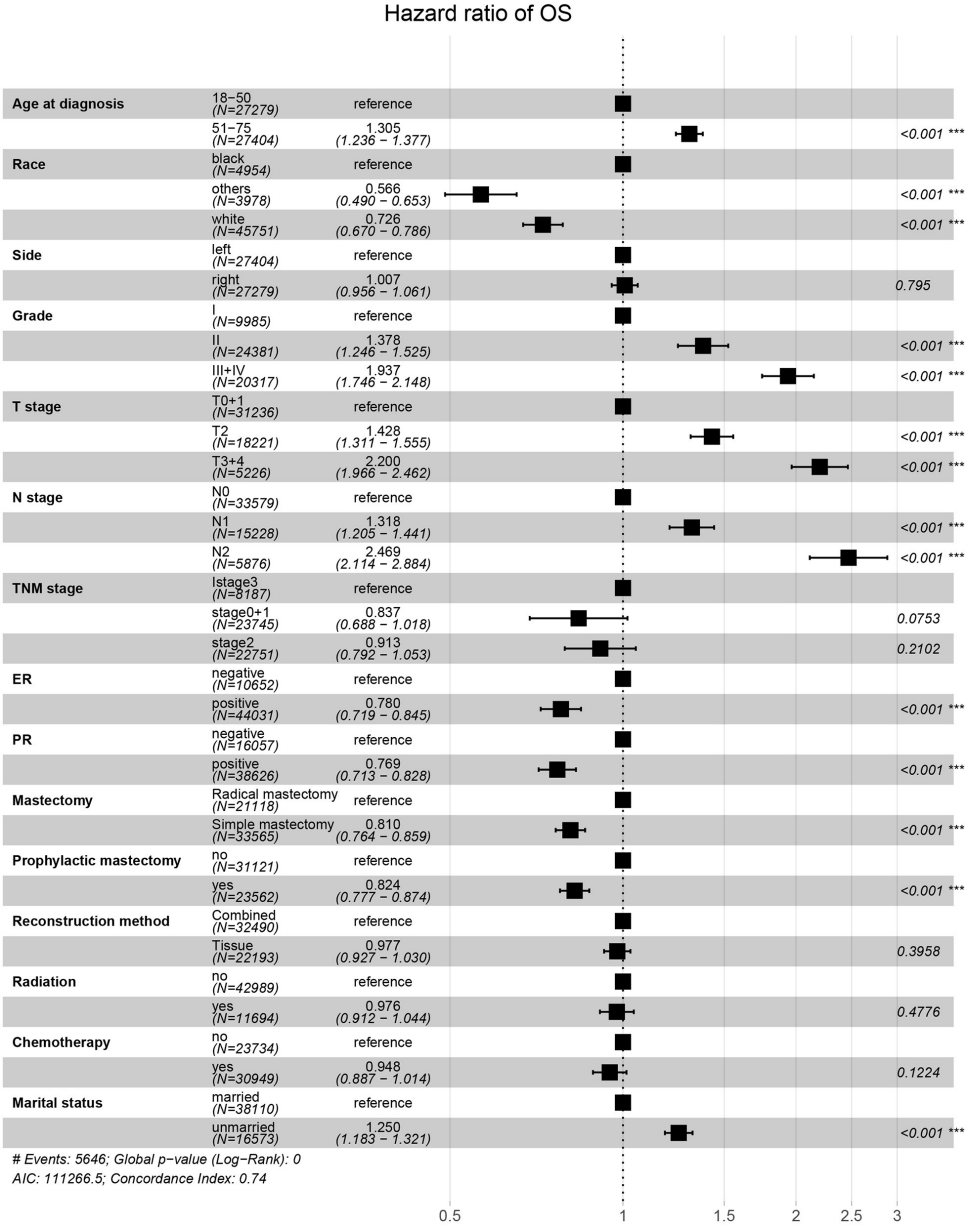
Forest plot.

To investigate the independent prognostic factors in OS, a multivariate Cox regression model was performed (Table 2). Patients in the unmarried group had poorer OS than those in the married group (HR: 1.250, 95% CI: 1.183–1.321). In addition, our results also showed that for female patients with breast reconstruction, older age, black race, higher histological grade, T2 stage and T3–T4 stage and N1 stage and N2 stage, ER negative, and PR negative tumors were considerably correlated with worse OS prognosis, while prophylactic mastectomy was correlated with better OS prognosis. A forest plot of HRs was generated to illustrate the prognostic factors in OS (Figure 3). After PSM, patients in the unmarried group still had worse OS than those in the married group (HR: 1.247, 95% CI: 1.148–1.355).

**Table 2:** Multivariate Cox regression model analysis of OS.

	**before PSM**				**after PSM**			
**Characteristics**	**HR**	**95% CI**		***P* value**	**HR**	**95% CI**		***P* value**
Age at diagnosis								
18–50								
51–75	1.304	1.236	1.376	0.000	1.372	1.257	1.496	0.000
Race								
White				0.000				
Black	1.377	1.272	1.491	0.000	1.494	1.286	1.736	0.000
Others	0.780	0.687	0.885	0.000	0.583	0.411	0.828	0.003
Side								
Left								
Right	1.007	0.956	1.061	0.793	0.977	0.899	1.061	0.579
Grade								
I								
II	1.378	1.246	1.524	0.000	1.354	1.159	1.582	0.000
III + IV	1.936	1.745	2.147	0.000	1.994	1.690	2.353	0.000
T stage								
T0 + T1								
T2	1.427	1.311	1.555	0.000	1.481	1.277	1.717	0.000
T3 + T4	2.196	1.963	2.458	0.000	2.323	1.893	2.852	0.000
N stage								
N0								
N1	1.317	1.205	1.440	0.000	1.404	1.210	1.630	0.000
N2	2.464	2.109	2.878	0.000	2.510	1.879	3.354	0.000
TNM stage								
0 + 1								
2	1.091	0.969	1.228	0.150	0.921	0.757	1.119	0.408
3	1.195	0.983	1.454	0.074	1.030	0.722	1.468	0.872
ER								
Positive								
Negative	1.282	1.182	1.390	0.000	1.153	0.986	1.348	0.075
PR								
Positive								
Negative	1.301	1.207	1.401	0.000	1.276	1.107	1.472	0.001
Mastectomy								
Simple mastectomy								
Radical mastectomy	1.234	1.163	1.309	0.000	1.275	1.158	1.404	0.000
Prophylactic mastectomy							
No								
Yes	0.824	0.777	0.874	0.000	0.806	0.730	0.890	0.000
Reconstruction method								
Tissue								
Implant	1.023	0.966	1.083	0.446	1.050	0.960	1.150	0.285
Combined	1.025	0.947	1.109	0.540	1.115	0.974	1.275	0.114
Radiation								
No								
Yes	0.976	0.912	1.045	0.482	0.990	0.873	1.123	0.879
Chemotherapy								
No								
Yes	0.949	0.887	1.015	0.124	0.896	0.801	1.002	0.055
Marital status								
Married								
Unmarried	1.250	1.183	1.321	0.000	1.247	1.148	1.355	0.000

### The Competing Risk Mode Analysis of BCSD and OCSD

**Figure 4:**
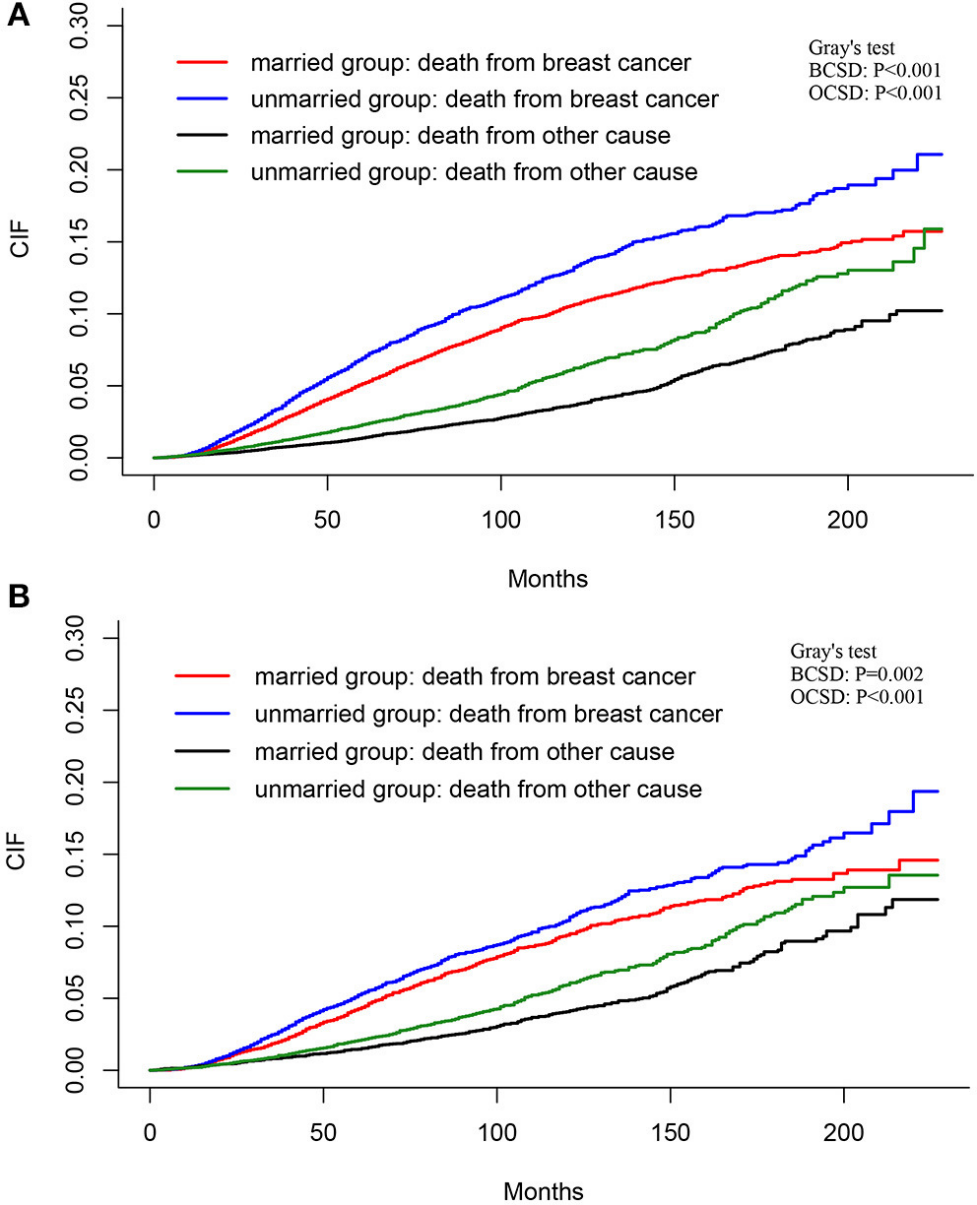
Cumulative incidence estimates of BCSD and non-BCSD of patients before **(A)** and after **(B)** PSM. BCSD, breast cancer-specific death; PSM, propensity score matching.

The total cumulative incidence of BCSD was as high as 7.32% (4,003/54,683), but the cumulative OCSD incidence was 3.00% (1,643/54,683). The total cumulative incidence of BCSD accounted for 6.80% (2,590/38,110) in the married group, and 8.53% (1,413/116,573) in the unmarried group. As shown in Figure 4A, the cumulative BCSD and OCSD rates at 5 years are 5.16 and 1.38% for patients in the married group, respectively, while 6.92 and 2.30% for those in unmarried group, respectively. Patients in the married group had better cumulative BCSD incidence (Gray's test, *p* < 0.001) and OCSD incidence (Gray's test, *p* < 0.001) than those in the unmarried group (Gray's test, *p* < 0.001).

As shown in Figure 4B, after PSM, the total BCSD and OCSD are as high as 6.07 and 2.94%, with the 5-year BCSD rate 4.27% for married patients and 5.23% for the unmarried group, respectively, and 5-year OCSD rate 1.46% for married patients and 2.07% for unmarried group, respectively. Compared with the unmarried group, patients in married group tended to have lower cumulative BCSD (Gray's test, *p* = 0.002) and OCSD (Gray's test, *p* < 0.001) incidences.

### Multivariate Competing Risk Regression Model Analysis

To further investigate the independent prognostic factors in BCSD, a multivariate competing risk regression model analysis was performed (Table 3). Patients in the unmarried group had worse BCSD (HR: 1.172, 95% CI: 1.094–1.254, *p* < 0.001) and OCSD (HR: 1.416, 95% CI: 1.281–1.566, *p* < 0.001) than those in the married group. In addition, patients in the radical mastectomy subgroup, T3 + T4 stage subgroup, Black race subgroup, had worse BCSD and OCSD than those in the corresponding subgroups.

**Table 3:** Competing risk model analysis of BCSD and non-BCSD.

	**Before PSM**										**After PSM**									
	**Cancer-specific death**					**Death from other causes**					**Cancer-specific death**					**Death from other causes**				
**Characteristics**	**Coefficient**	**HR**	**95% CI**		***P*** **value**	**Coefficient**	**HR**	**95% CI**		***P*** **value**	**Coefficient**	**HR**	**95% CI**		***P*** **value**	**Coefficient**	**HR**	**95% CI**		***P*** **value**
Age at diagnosis																				
18–50																				
51–75	−0.003	0.997	0.934	1.064	0.920	0.911	2.487	2.234	2.770	0.000	0.018	1.0180	0.9140	1.1340	0.750	0.916	2.5000	2.1200	2.9490	0.000
Race																				
White																				
Black	0.311	1.364	1.239	1.503	0.000	0.230	1.258	1.071	1.479	0.005	0.374	1.4530	1.2050	1.7520	0.000	0.307	1.3590	1.0230	1.8040	0.034
Others	−0.224	0.799	0.690	0.925	0.003	−0.306	0.736	0.572	0.947	0.017	−0.348	0.7060	0.4700	1.0600	0.093	−0.943	0.3900	0.1940	0.7810	0.008
Side																				
Left																				
Right	0.021	1.021	0.959	1.088	0.510	−0.029	0.971	0.882	1.070	0.550	−0.011	0.9890	0.8910	1.0980	0.840	−0.037	0.9640	0.8370	1.1090	0.610
Grade																				
I																				
II	0.577	1.780	1.539	2.060	0.000	0.113	1.119	0.973	1.288	0.120	0.665	1.9440	1.5010	2.5180	0.000	0.130	1.1390	0.9340	1.3890	0.200
III + IV	1.037	2.822	2.433	3.274	0.000	0.085	1.089	0.929	1.276	0.290	1.253	3.5020	2.6870	4.5630	0.000	0.063	1.0650	0.8360	1.3570	0.610
T stage																				
T0 + T1																				
T2	0.410	1.507	1.361	1.668	0.000	0.099	1.104	0.930	1.312	0.260	0.428	1.5340	1.2790	1.8400	0.000	0.199	1.2210	0.9270	1.6070	0.160
T3 + T4	0.839	2.313	2.027	2.640	0.000	0.303	1.354	1.044	1.756	0.022	0.865	2.3740	1.8620	3.0280	0.000	0.437	1.5480	0.9760	2.4530	0.063
N stage																				
N0																				
N1	0.301	1.351	1.213	1.505	0.000	0.105	1.111	0.927	1.331	0.250	0.309	1.3620	1.1360	1.6320	0.001	0.309	1.3610	1.0270	1.8040	0.032
N2	0.981	2.668	2.218	3.210	0.000	0.250	1.285	0.893	1.847	0.180	0.963	2.6200	1.8570	3.6960	0.000	0.388	1.4740	0.7790	2.7870	0.230
TNM stage																				
0 + 1																				
2	0.264	1.302	1.123	1.510	0.000	0.014	1.014	0.813	1.266	0.900	0.079	1.0820	0.8430	1.3890	0.530	−0.184	0.8320	0.5870	1.1790	0.300
3	0.367	1.444	1.138	1.833	0.003	0.142	1.153	0.749	1.775	0.520	0.223	1.2500	0.8130	1.9200	0.310	−0.109	0.8970	0.4230	1.9020	0.780
ER																				
Positive																				
Negative	0.262	1.300	1.177	1.436	0.000	0.157	1.170	0.992	1.380	0.063	0.127	1.1350	0.9290	1.3860	0.210	0.098	1.1030	0.8340	1.4600	0.490
PR																				
Positive																				
Negative	0.349	1.417	1.292	1.553	0.000	0.005	1.005	0.871	1.160	0.950	0.311	1.3650	1.1340	1.6420	0.001	0.073	1.0750	0.8490	1.3620	0.550
Mastectomy																				
Simple mastectomy																				
Radical mastectomy	0.173	1.189	1.107	1.277	0.000	0.298	1.347	1.210	1.499	0.000	0.232	1.2610	1.1160	1.4260	0.000	0.266	1.3040	1.1180	1.5220	0.001
Prophylactic mastectomy																			
No																				
Yes	−0.176	0.839	0.782	0.900	0.000	−0.290	0.749	0.668	0.839	0.000	−0.234	0.7910	0.7000	0.8940	0.000	−0.232	0.7930	0.6680	0.9420	0.008
Reconstruction method																			
Tissue																				
Implant	−0.068	0.934	0.871	1.002	0.056	0.203	1.225	1.102	1.363	0.000	−0.034	0.9670	0.8630	1.0830	0.560	0.162	1.1750	1.0080	1.3700	0.039
Combined	0.010	1.010	0.918	1.112	0.830	0.063	1.065	0.918	1.235	0.410	0.142	1.1530	0.9730	1.3650	0.100	0.023	1.0230	0.8110	1.2900	0.850
Radiation																				
No																				
Yes	0.039	1.040	0.958	1.129	0.350	−0.238	0.788	0.679	0.915	0.002	−0.013	0.9870	0.8480	1.1490	0.860	−0.028	0.9720	0.7470	1.2660	0.830
Chemotherapy																				
No																				
Yes	0.123	1.130	1.035	1.235	0.007	−0.306	0.736	0.656	0.827	0.000	0.176	1.1930	1.0220	1.3910	0.025	−0.474	0.6220	0.5170	0.7490	0.000
Marital status																				
Married																				
Unmarried	0.158	1.172	1.094	1.254	0.000	0.348	1.416	1.281	1.566	0.000	0.167	1.1820	1.0650	1.3120	0.002	0.303	1.3540	1.1750	1.5600	0.000

*BCSD, breast cancer-specific death*.

After PSM, participants in the unmarried group still had worse BCSD (HR: 1.182, 95% CI: 1.065–1.312, *p* = 0.002) and OCSD (HR: 1.354, 95% CI: 1.175–1.560, *p* < 0.001) than those in the married group.

### Survival Analysis of Marital Status in Four Molecular Subgroups

**Figure 5:**
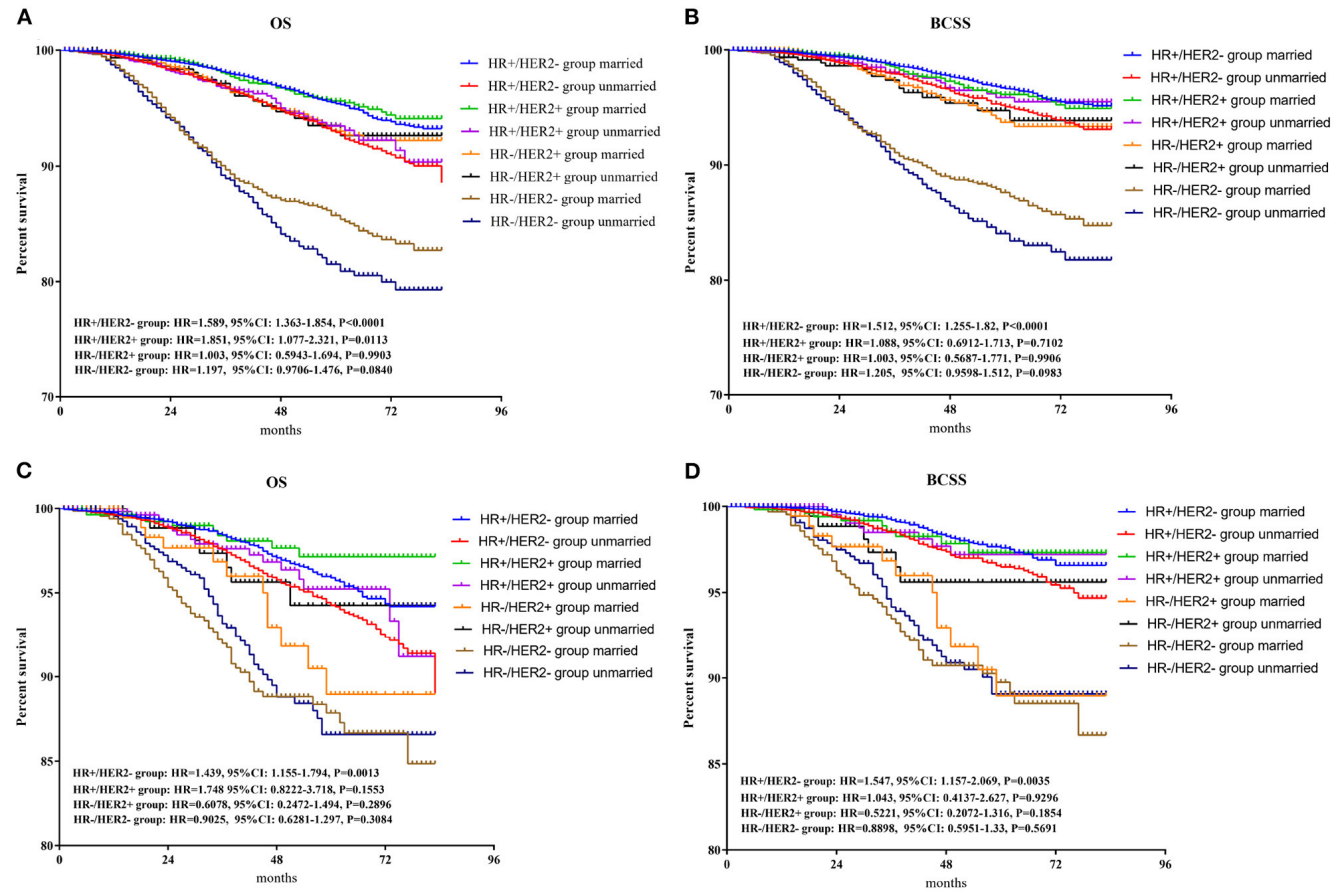
Survival analysis of OS and BCSS between married and unmarried patients in HR+/HER2 +, HR+/HER2–, HR–/HER2 +, and HR–/HER2– subgroups. BCSS, breast cancer-specific survival. **(A)** Overall survival for patients before PSM; **(B)** breast cancer-specific survival for patients before PSM; **(C)** overall survival for patients after PSM; **(D)** breast cancer-specific survival for patients after PSM. PSM, propensity score matching.

To further explore the survival prognosis of marital status in different molecular types, Kaplan-Meier analyses were performed. As shown in in Figure 5A, patients in the unmarried group show significantly or borderline worse OS prognosis than those in the married group with HR+/HER2– (HR = 1.589, 95% CI: 1.363–1.854, *p* < 0.001), HR+/HER2 + (HR = 1.851, 95% CI: 1.077–2.321, *p* = 0.0113), and HR–/HER2– (HR = 1.179, 95% CI: 0.971–1.476, *p* = 0.084) breast cancers. However, no significant differences in OS were found between the unmarried and married groups for patients with HR–/HER2 + breast cancer. As shown in Figure 5B, patients in the unmarried group have poorer or borderline BCSS than those in the married group with HR+/HER2– (HR =1.512, 95% CI: 1.255–1.82, *p* < 0.001) breast cancer or HR–/HER2– (HR = 1.205, 95% CI: 0.960–1.512, *p* = 0.0983), while no significant differences between the married and unmarried groups were found for patients with HR+/HER2 + or HR–/HER2 + breast cancers. After PSM, as shown in Figures 5C,D, both OS and BCSS benefits are found between the married and unmarried groups for patients with HR+/HER2– breast cancer (OS: HR = 1.439, 95% CI: 1.155–1.794, *p* = 0.0013; BCSS: HR = 1.547, 95% CI: 1.157–2.069, *p* = 0.0035), while, there were no differences of OS or BCSS prognosis between married and unmarried patients in other three subgroups.

## Discussion

In the current study, we investigated the relationship between the long-term survival prognosis and marital status for patients with female breast cancer (FBC) who underwent breast reconstruction. Based on the analysis of a large cohort of 54,683 patients with FBC after breast reconstruction in the SEER database from 1998 to 2015, we could confirm that married patients had better both OS and BCSS than unmarried patients, by using an integrated range of factors into a competing risk regression model and PSM method.

Clinicopathological features, such as age, grade, tumor stage, molecular subtype, have been considered as reliable factors predicting the prognosis of patients and guiding clinical therapy for patients with FBC (14–16). Higher differentiation level, T stage and N stage, ER negative, and PR negative tumors were regarded as an adverse prognostic factor predicting OS and BCSS (15, 17, 18). Similar to previous trials (15, 17, 18), in the current study, older age, higher differentiation level, T stage and N stage, and negative ER and PR statuses were considerably correlated with worse OS prognosis. Therefore, our findings confirm that patients with breast reconstruction have multiple negative features related to poor outcomes.

As one of the social and psychological aspects which could influence the prognosis of breast cancer patients, marital status has attracted increasing attention in the breast cancer field. In the current study, 69.69% (38,110) subjects were married, 30.31% (16,573) were not married. Compared with the unmarried group, the married group appeared to be associated with both improved OS and BCSS after Kaplan-Meier analysis. The HRs of 1.250 (95% CI: 1.183–1.321) imply that marriage was an independent prognostic factor for FBC with reconstructed breast through Cox proportional hazard analysis. Such results indicated that marriage tended to prolong the long-term survival for patients with FBC who underwent breast reconstruction. However, the competing risk, which could disturb BCSD and hamper the emergence of the primary event attributed to the estimation bias arising from OCSD, should not be neglected.

To undermine the underlying estimation bias of BCSD, which had been recognized as one the most valuable prognostic indexes for breast cancer patients (17, 19) and further investigate the efficiency of marital status on patients with reconstructed breast, the Fine and Gray competing risk model and multivariable competing risk regression model were utilized. The results showed that the inexistence of marriage was associated with worse BCSD and OCSD for FBC patients with breast reconstruction. The benefit arising from marriage was consistent with previous reports, which showed that the presence of marriage was correlated with a better prognosis for participants with breast cancer patients (9, 10). The underlying reason may be that the existence of marriage means more financial and emotional supports and then promotes the long-term prognosis. Consequently, the existence of marriage plays a vital part in decreasing BCSD and OCSD and should be a choice of the surgical treatment regimen for FBC patients with breast reconstruction.

Breast cancer could be divided into 4 molecular subtypes categorized according to ER or PR expression and HER2 gene amplification, each molecular subtype of breast cancer means a different distinct risk profile and treatment strategy (19–21). To investigate the effect of molecular subtypes on survival, a subgroup analysis was applied. In the HR+/HER2- subgroup, married patients had both improved OS and BCSS in comparison with unmarried patients. And in the HR+/HER-2+ subgroup, improved OS was only found in married patients. No significant differences in OS or BCSS were found between married and unmarried subjects in HR-/HER2+ or HR-/HER2– subgroups. The underlying reason may be that hormone exposure would accelerate (hormone receptor positive) HR + breast cancer proliferation, and estrogen and progestin would therefore be risk factors for breast cancer (22–25). As marital status may affect the gonadal hormone level in women, we supposed that marital status may influence the OS of HR + patients through interacting with HR. Interestingly, after PSM, only married patients in HR+/HER2– subgroup showed a better survival prognosis than unmarried in both OS and BCSS after PSM. It seems that married status may affect the survival of breast cancer patients through HR, which should be proved by further experiments.

## Conclusion

In summary, this research demonstrated that the existence of marriage could reduce the risk of BCSD for female patients undergoing breast reconstruction, especially for patients with HR+/HER2– molecular subtype. In view of our study's results, we conclude that the inexistence of marriage was associated with poorer OS and BCSS, especially for HR+/HER2– subtype breast cancer women undergoing breast reconstruction. Large randomized controlled clinical trials are still needed to further provide a high level of evidence on prognostic value of married status for patients with reconstructed breast.

## Data Availability Statement

Publicly available datasets were analyzed in this study. This data can be found here: All data and materials analyzed during this study are available from the SEER database.

## Author Contributions

CZ and JL: study concept and design, critical revision of the manuscript, obtained funding, and study supervision. SZ, ZY, and PQ: drafting of the manuscript. SZ, ZY, PQ, and JL: statistical analysis. All authors contributed to the article, acquisition, analysis, or interpretation of data, and approved the submitted version.

## Funding

This work was supported by the National Natural Science Foundation of China (Grant No: 81502413) and Shaanxi Provincial Natural Science Foundation of China (Grant No.2019SF-145).

## Conflict of Interest

The authors declare that the research was conducted in the absence of any commercial or financial relationships that could be construed as a potential conflict of interest.

## Publisher's Note

All claims expressed in this article are solely those of the authors and do not necessarily represent those of their affiliated organizations, or those of the publisher, the editors and the reviewers. Any product that may be evaluated in this article, or claim that may be made by its manufacturer, is not guaranteed or endorsed by the publisher.
